# The Development of a Novel High Throughput Computational Tool for Studying Individual and Collective Cellular Migration

**DOI:** 10.1371/journal.pone.0082444

**Published:** 2013-12-27

**Authors:** Douglas A. Chapnick, Jeremy Jacobsen, Xuedong Liu

**Affiliations:** 1 Department of Chemistry and Biochemistry, University of Colorado, Boulder, Colorado, United States of America; University of Birmingham, United Kingdom

## Abstract

Understanding how cells migrate individually and collectively during development and cancer metastasis can be significantly aided by a computation tool to accurately measure not only cellular migration speed, but also migration direction and changes in migration direction in a temporal and spatial manner. We have developed such a tool for cell migration researchers, named Pathfinder, which is capable of simultaneously measuring the migration speed, migration direction, and changes in migration directions of thousands of cells both instantaneously and over long periods of time from fluorescence microscopy data. Additionally, we demonstrate how the Pathfinder software can be used to quantify collective cell migration. The novel capability of the Pathfinder software to measure the changes in migration direction of large populations of cells in a spatiotemporal manner will aid cellular migration research by providing a robust method for determining the mechanisms of cellular guidance during individual and collective cell migration.

## Introduction

Cellular migration has been shown to be an important process in cancer progression, development, tissue repair, and immune response [1]–[10]. As a result, a plethora of research has been performed to identify the molecular mechanisms behind how individual cells achieve migration, as well as how neighboring cells migrate cooperatively in collective migration (reviewed in [11]–[13] and [14], respectively). Collective migration is defined as the ability of physically interacting cells to adopt a common migration direction [14], [15]. Like individual cell migration, the collective migration of cells has been shown to be an important process in cancer progression, development and wound repair [16]–[23]. Such collective behavior results from each cell responding to the environmental stimuli of neighboring cells, in addition to non-cell environmental stimuli [4], [5], [14], [15], [17], [19], [20], [24]–[32]. Although a relatively large amount of research has been conducted to determine mechanisms behind individual cell migration, far less is known about exactly how cells migrate collectively. Furthermore, there is no standard method in the literature to quantify the ‘collectiveness’ behavior during collective migration [33]–[35].

Previous research into individual cell migration has revealed important fundamental mechanisms by which cells migrate. For instance, when an individual cell migrates on a two-dimensional (2D) surface, it projects a front end extension that can either be broad (termed a llamelipodia) or with multiple spike-like extensions (termed fillipodia), which are the result of coordinated polymerization, depolymerization, and branching of the actin cytoskeleton [12],[24],[36]–[46]. Such coordination of actin dynamics is controlled by local recruitment of cell polarity maintain proteins, such as CDC42/Rac and Rho, which either directly or indirectly regulate actin structure, polymerization, and attachment to the extracellular matrix [37], [38], [47]–[51]. The attachment of the actin cytoskeleton is largely mediated by protein complexes, termed focal adhesions, which anchor the actin cytoskeleton to trans membrane integrin receptors and the extracellular matrix [52]. The assembly of focal adhesions allows for the cell to successfully attach a front end extension to the extracellular matrix and the disassembly of focal adhesions allows a cell to detach the rear during rear end retraction [53]–[55]. Focal adhesion turnover and the resulting changes to the actin cytoskeleton are regulated by several kinase activities, including focal adhesion kinase (FAK), Src kinase and Rho GTPase [12], [56]–[60]. The temporal and spatial regulation of both the actinomyosin skeleton and focal adhesions are regulated by a complex combination of growth factor signaling and extracellular matrix protein activities, which influence the speed of actin and focal adhesion dynamics, ultimately influencing how fast a cell can migrate [61], [62].

Our current understanding of the biochemical mechanisms underlying cellular migration have been primarily the result of *in vitro* studies conducted in 2D cell culture model systems [2], [11]–[13], [24], [31], [32], [38], [40]–[43], [54], [56], [57], [60], [62]–[70]. However, several critical biochemical activities governing cell migration have proven to play similar roles in three dimensional (3D) model systems and *in vivo*. For instance, in 2D, 3D and *in vivo* experiments, CDC42/Rac activity determine cellular polarity [71], [72]. Similarly, FAK kinase mediates cellular migration both in 2D and 3D assays [73]–[75]. As a result, investigations performed in 2D assays have shed light on biochemical mechanisms that have proven to have physiological relevance. However, recent research has also revealed that there is significant difference in cell migration machinery between cells in 2D versus 3D [75]–[77]. Although the conclusions made in 2D migration studies will always require confirmation of physiological relevance in *in vivo* studies, they remain a valuable tool for initial investigations into the molecular mechanisms behind cellular migration compared to 3D and *in vivo* studies because they allow for tight control of experimental conditions and more accurate observation of cellular migration behavior at single cell resolution without the use of relatively complex microscopes, such as two-photon and confocal microscopes. Many of the concerns about discrepancies in biochemical mechanisms behind 2D and 3D motility may prove to be overcome by imaging individual cell motility in 2D on soft extracellular matrices, which have been shown to be more closely similar to *in vivo* tissues than plastic or glass cell culture plates [78].

The behavior of migrating cells can be characterized by migration speed, migration direction, and migration persistence (the ability of a cell to maintain its migration direction). In 2D studies, the measurement of cell migration behavior is conducted by either manual cell tracking [79]–[81] or automated cell tracking [31], [32], [61], [82]–[85]. Such cell tracking experiments have not only shed light on how a cell achieves migration, but also have shown that cells can undergo chemotaxis towards a localized biochemical signals [20], [86], [87]. In these studies, a Dunn Chamber is used to present a chemokine gradient to cells, where cells migrate upstream of Epidermal Growth Factor (EGF) and Urokinase Plasminogen Activator (uPA) gradients [88]. Such studies into how cells achieve chemotaxis highlight the need for cell migration tracking programs to not only calculate the speed and persistence of cells, but also to report the direction and changes in direction during cellular migration.

**Figure 1:**
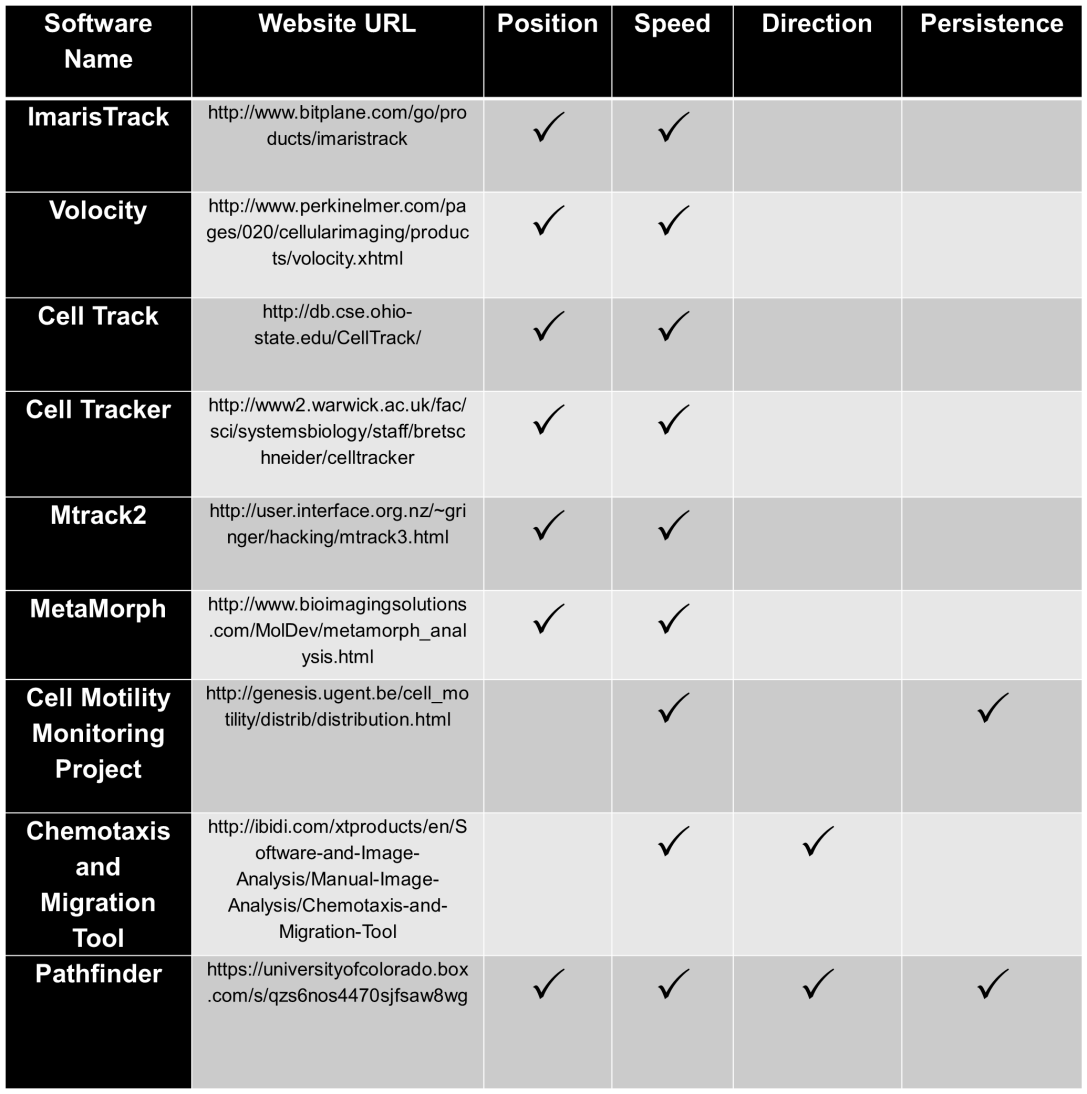
The Pathfinder cell motility program uniquely incorporates measurements of cellular position, speed, direction, and persistence. Although several cellular motility programs are available to measure cellular motility in terms of position and speed, only the Pathfinder program additionally reports both cellular migration direction and cellular migration persistence.

Although several computational tools exist that allow for automated cell tracking of individual cells in time-lapse microscopy videos (**Fig. 1**), these tools focus almost entirely on either the speed or persistence (ability to maintain a migration direction) or a cell. We have developed an automated high throughput cell tracking software, named Pathfinder, which is capable of simultaneously measuring and reporting cellular migration speed, migration direction and changes in migration direction of thousands of fluorescently labeled cells for an unlimited number of microscopy videos. The Pathfinder software has two improved features that distinguish it from previous cell motility computational tools, as the Pathfinder output is able to report instantaneous cell migration direction and changes in cellular migration direction. Although the cellular migration field has elucidated many fundamental mechanisms behind cellular migration, there are several key questions that remain to be answered. Specifically, ‘how does a cell select a migration direction?’ and ‘what makes a cell change direction?’ Answering these questions will require the ability to study the migration direction and changes in migration direction at single cell and instantaneous resolution. Additionally, we explain how measurements of cellular migration direction can be used to quantify collective migration. In summary, the Pathfinder software aims to propel the cell migration field forward in both the mechanistic understanding of individual cell migration, as well as collective cell migration by allowing researchers to fully characterize cell migration behavior in an automated and high throughput manner.

## Methods

### Fluorescent Labeling of Cells, Cell Culture and Cellular Imaging

Stable transgenic HaCaT (Cell Lines Services, Germany) and MDA-MB-231 (ATCC, HTB-26) cell lines were fluorescently labeled via retroviral mediated gene transfer of mCherry-Histone H2B using the pRex-mCherry-H2B plasmid. For all experiments, cells were cultured in DMEM lacking phenolphthalein red and supplemented with 2 mM L-glutamine, 100 Units/mL penicillin and 100 µg/mL streptomycin. For low density assays, cells were plated at an average density of 300 cells/mm^2^ for both HaCaT and MDA-MB-231 cells. For confluent monolayer experiments, HaCaT cells were plated at an average density of 1000 cells/mm^2^. For epithelial sheet assays, HaCaT cells were plated at an average density of 1200 cells/mm^2^ for 3 hours at 37°C, after which half of partially adherent cells were manually removed using a 200 µl pipet tip. Transforming Growth Factor-Beta (TGFβ) stimulations were conducted with 100 pM ligand, while EGF stimulations were conducted with 100 nM ligand. An ImageXpress MicroXL high throughput wide-field fluorescence microscope (Molecular Devices) was used for imaging experiments at 37°C and 5% CO_2_. All microscopy videos were acquired with a frame rate of one frame every seven minutes, in the mCherry fluorescence channel at a magnification of 10×, where each pixel represents 1.314 µm at a pixel binning of 2×2. The accompanying MetaXpress software was used to compile video files from time-lapse images for each well of a 96 well plate.

### Programming, Input Parameters, and Output for the Pathfinder Software

The Pathfinder software (https://universityofcolorado.box.com/s/qzs6nos4470sjfsaw8wg and **Data S1**) was written in the Java programming language. User specification is required for cell radius (pixels), minimum track length, the interval of frames for desired calculations (frame n –frame n+a, where a represents the number of frames to skip for calculations), percentage of pixels in the video that represent cells, and the directory path for the folder containing .avi files (**Fig. S1**). The output for each video file is a single Excel spreadsheet (**Fig. S2 and Data S2**). The Pathfinder software requires only decompression of the attached .zip file and installation of JAVA runtime environment on either a 32-bit or 64-bit Windows Machine. Please note that use of pathfinder on a 64-bit machine allows for higher memory use in Java, which allows for analysis of greater numbers of cells in a single video.

### Calculation of Migration Parameters, Persistence Time and Nearest Neighbor Analyses

Cellular speed was calculated as the displacement of a cell (pixels) over 1 frame. Conversion to µm/hour is determined by the following equation:
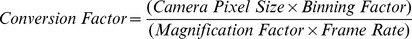



The Angle of Trajectory was calculated from the following discontinuous equations:
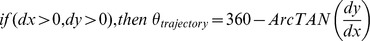


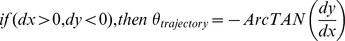


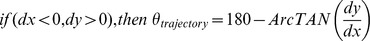


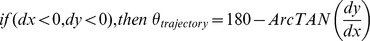


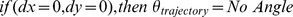


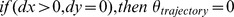


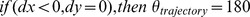


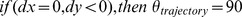


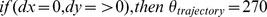



Angle of Deflection was calculated from the following discontinuous equations:
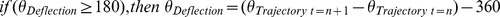


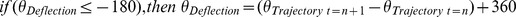



Persistence time calculations were performed using a modified in-house MatLab based program developed by Dr. Douglas Lauffenburger (MIT) (**Data S3**).

Nearest neighbor calculations were done in Excel using an inter-centroid distance matrix of (all cells)×(all cells) for each frame. Nearest neighbors were defined as cells whose centroids are within 100 µm of each other. Paired random migration index of the angle of trajectory (PRMI Θ_Trajectory_) calculations were also done in Excel, where the standard deviation of the migration directions between pairs of neighboring cells were averaged over greater than 100 pairs of cells for each condition. This average of standard deviations is referred to as the PRMI Θ_Trajectory_.

## Results

### The overview and capabilities of the pathfinder software

**Figure 2:**
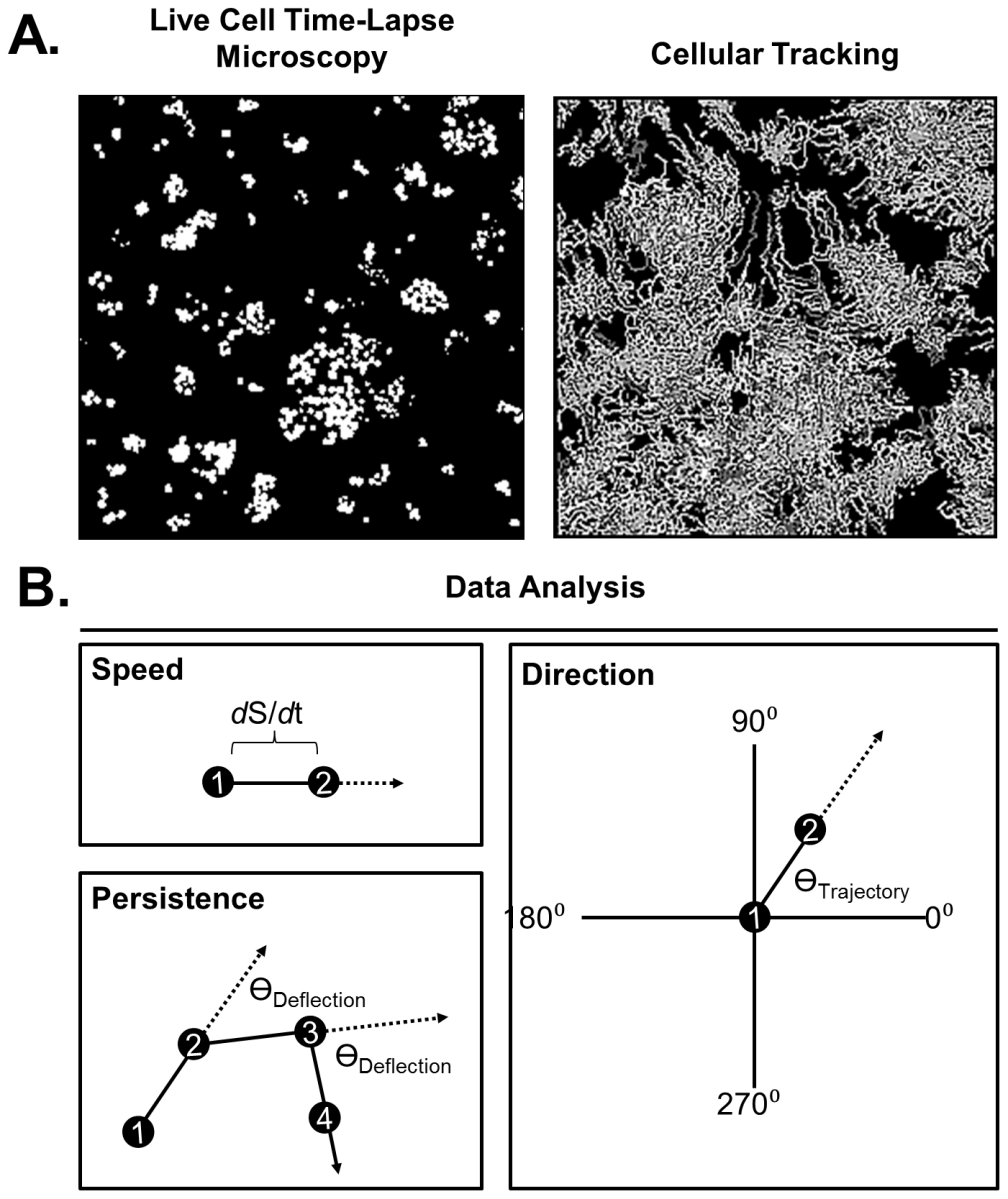
Angular measurements of cellular migration can reveal cellular behavior. **A**) The Pathfinder program converts time-lapse microscopy videos of fluorescent HaCaT cells (left) to cellular tracks (right). **B**) The Pathfinder program uses the positional information of cellular tracks to calculate speed, where *d*S represents change in position and *d*t represents change in time (top), migration persistence through the absolute angle of deflection (bottom), and the migration direction relative to a well-defined axis orientation in the field of view (right).

The JAVA based Pathfinder software was developed to allow researchers to easily analyze large data sets of time-lapse fluorescence microscopy videos of motile cells. Since cellular tracking is already a well-established technique, our software implements a previously validated tracking algorithm (‘Particle Tracker’) developed by Sbalzarini *et. al* to detect each fluorescently labeled nuclei in each frame (**Fig. 2A** **, left**), as well as to assemble such positional information into cellular tracks (**Fig. 2A** **, right**), as described in their publication [89]. Since cellular positions alone are of little use to researchers in the cell migration field, we developed an analysis algorithm to transform the previous ‘Particle Tracker’ output into an excel spreadsheet that displays calculations of the speed, the direction, and changes in direction of individual cells, as well as the average values for a population of cells (**Fig. 2B** **, S3, and S4**). Simultaneous reporting of these parameters makes the Pathfinder software unique compared to other available computational motility programs (**Fig. 1**). In addition, Pathfinder is capable of running batch parallel processing of unlimited .avi files, allowing for automated and high throughput data processing of fluorescent time-lapse microscopy videos, provided they are placed in a single folder that can be navigated to from within the Pathfinder GUI. The Pathfinder program requires either a 32 bit or a 64 bit windows operating system with JAVA Runtime Environment.

### Fluorescent Labeling of Cells Does not Significantly Alter Cell Motility

We compared the migration speeds of wild type MDA-MB-231 cells to MDA-MB-231 cells expressing a nuclear fluorescence marker in the presence and absence of EGF in order to determine if the introduction of nuclear marker significantly impacted ligand induced migration. Unlabeled wild type cells were manual segmented and analyzed using Pathfinder, while fluorescent images were automatically segmented and analyzed using Pathfinder. Introduction of a fluorescent nuclear marker into these cells did not significantly alter the EGF induced cellular migration speed (**Fig. S3**). However, we do not rule out that different methods of gene delivery and types of nuclear markers (for instance a fluorescent protein other than histone H2B) could lead to permanent changes in cell migration.

### Using the average absolute angle of deflection to measure cellular persistence

**Figure 3:**
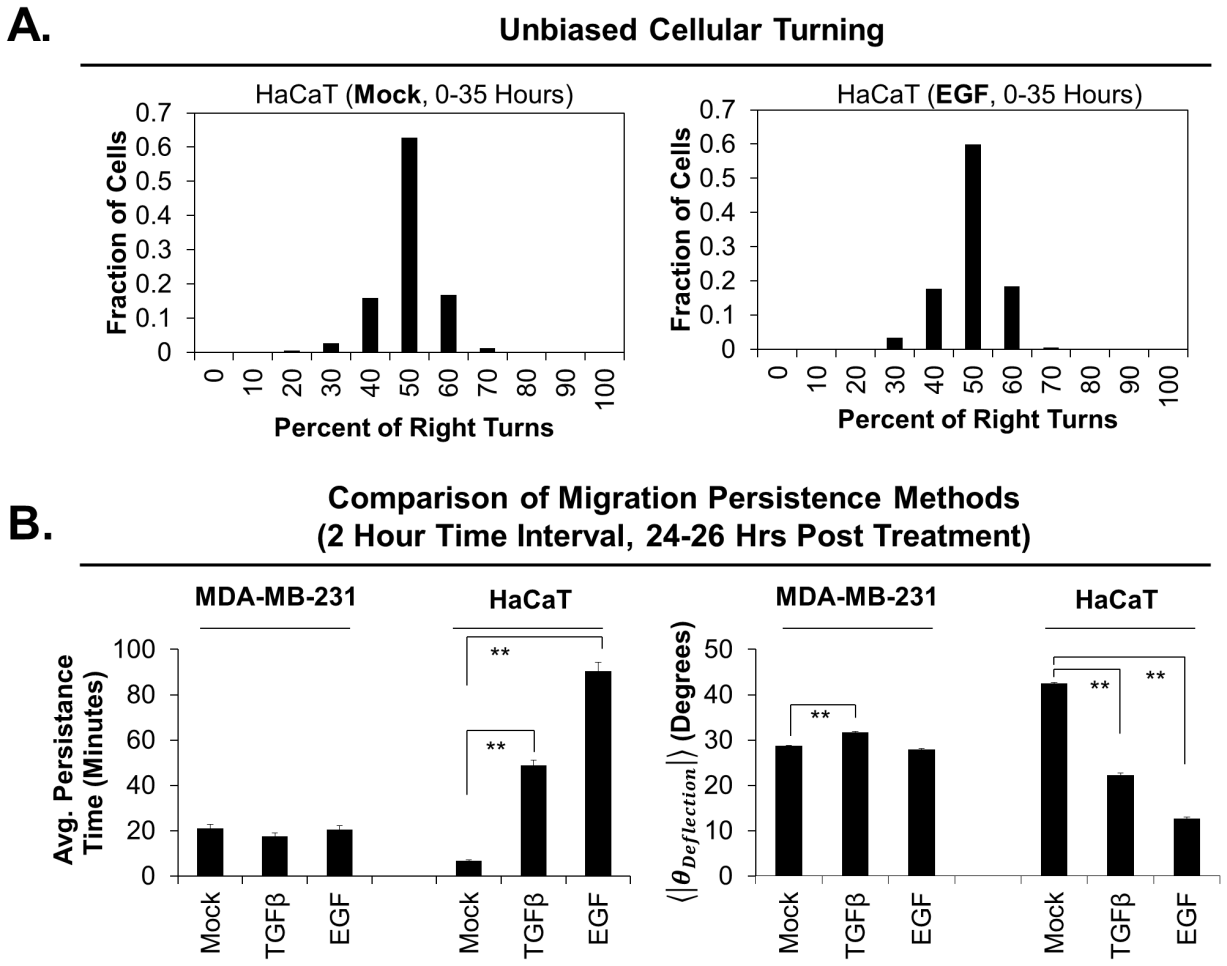
Average absolute angle of deflection measurements accurately depict the migration persistence of cells. **A**) Cells do not prefer to turn right or left in either the presence (right) or absence (left) of EGF stimulation. A binned histogram of percent of cells versus percent of right turns is normal and centered around 50 percent. **B**) A comparison of persistence time calculations and average absolute angle of deflection (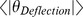) methods for measuring migration persistence yields identical trends in both the presence and absence of either TGFβ or EGF for MDA-MB-231 cells and HaCaT cells. Double asterisks indicate a *p* value<0.01. Each condition represents greater than 200 cells for persistence time measurements, and greater than 1000 cells for average absolute angle of deflection measurements.

In order to provide a means for high throughput calculation of cellular migration persistence, we used a non-traditional, but direct, approach of calculating the angle of deflection for each cell at each time. **Fig. 2B**, bottom left, illustrates how the angle of deflection measures migration persistence. The diagram represents a single cell, whose position is measured at three successive time points (1, 2, and 3, respectively). As the cell travels from 1 to 2 it maps out a line representing the trajectory of the cell between these two times. Similarly, as the cell travels from 2 to 3, another line is formed. The angle of deflection is the angle between these two lines, where a clockwise turn has a positive value, and a counterclockwise turn has a negative value. Using this calculation, each cell at each time can be assigned an angle of deflection, such that the sampling of many cells at a single time point can provide an accurate measurement of how straight cells are migrating within the population. A decrease in the average absolute value of the angle of deflection for a population of cells (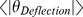) reflects an increase in the migration persistence. We use the absolute value of the angle of deflection for describing the persistence of migration in large populations, rather than maintaining the sign of the angle of deflection, because cells do not display a bias in which direction they prefer to turn (**Fig. 3A**).

### Comparing methods to measure migration persistence

Although we measure migration persistence using the average absolute angle of deflection, the measurement of migration persistence is currently conducted in the cell migration field through the determination of persistence time, which is calculated using data fitting of time dependent mean squared displacement trends to Equation 1, where *MSD* is the mean squared displacement of the cell, 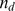 is the number of dimensions in which cells are migrating, 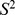 represents the squared speed of the cell, *P* represents the persistence time of a cell, and *t* is the time.
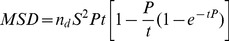
(1)


Persistence time is used to measure migration persistence because local changes in mean squared displacements trends are likely to be associated with changes in the direction of cellular migration, provided that speed is taken into consideration. A key difference between our approach and the persistence time approach is that persistence time measurements focus on how long a cell maintains a direction, while the average absolute angle of deflection measurements focus on the degree to which cells in a population turn in each frame. Thus, persistence time measurements reflect behavior over a specified interval of time (usually 2–4 hours), while the average absolute angle of deflection measurements reflect the relatively instantaneous behavior of cells.

We compared our technique of measuring migration persistence to the method of measuring persistence time by examining the time-lapse microscopy videos of two cell lines, MDA-MB-231 and HaCaT, stably expressing fluorescent nuclear markers and treated with no ligand, TGFβ or EGF. When such videos of HaCaT cells (24–26 hours post ligand stimulation) are analyzed by the two methods, both techniques lead to the same conclusions; TGFβ and EGF stimulation cause increased migration persistence, where EGF has an impact of higher magnitude than that of TGFβ (**Fig. 3B**). Both methods also agree when the same analysis is applied to MDA-MB-231 cells, where only TGFβ has a low magnitude effect on migration persistence (**Fig. 3B**). The measured persistence time of approximately 30 minutes observed for EGF stimulated MDA-MB-231 cells is consistent with similar results from other studies [82]. Since these two techniques yield the same results under these experimental conditions, we conclude that both techniques accurately measure migration persistence in motile cells. However, there is one critical difference between our method for measuring migration persistence compared to the persistence time method. Measuring the average absolute angle of deflection can yield a measurement for migration direction for each frame, while persistence time calculations require enough frames to construct a MSD vs time plot to fit to Equation 1. Thus, the persistence time calculation, which is traditionally calculated from sampling over 2–4 hours, cannot accurately report when a cell turns and how much it turns, but instead reports its average tendency to turn. The value of having the ability to measure exactly when an to what degree a cell turns will prove useful for future investigations into the mechanism by which cells are guided and will aid researchers in answering how exactly a cell determines where to extend its front end.

### Overlapping intervals suppresses noise in directional cellular behavior

**Figure 4:**
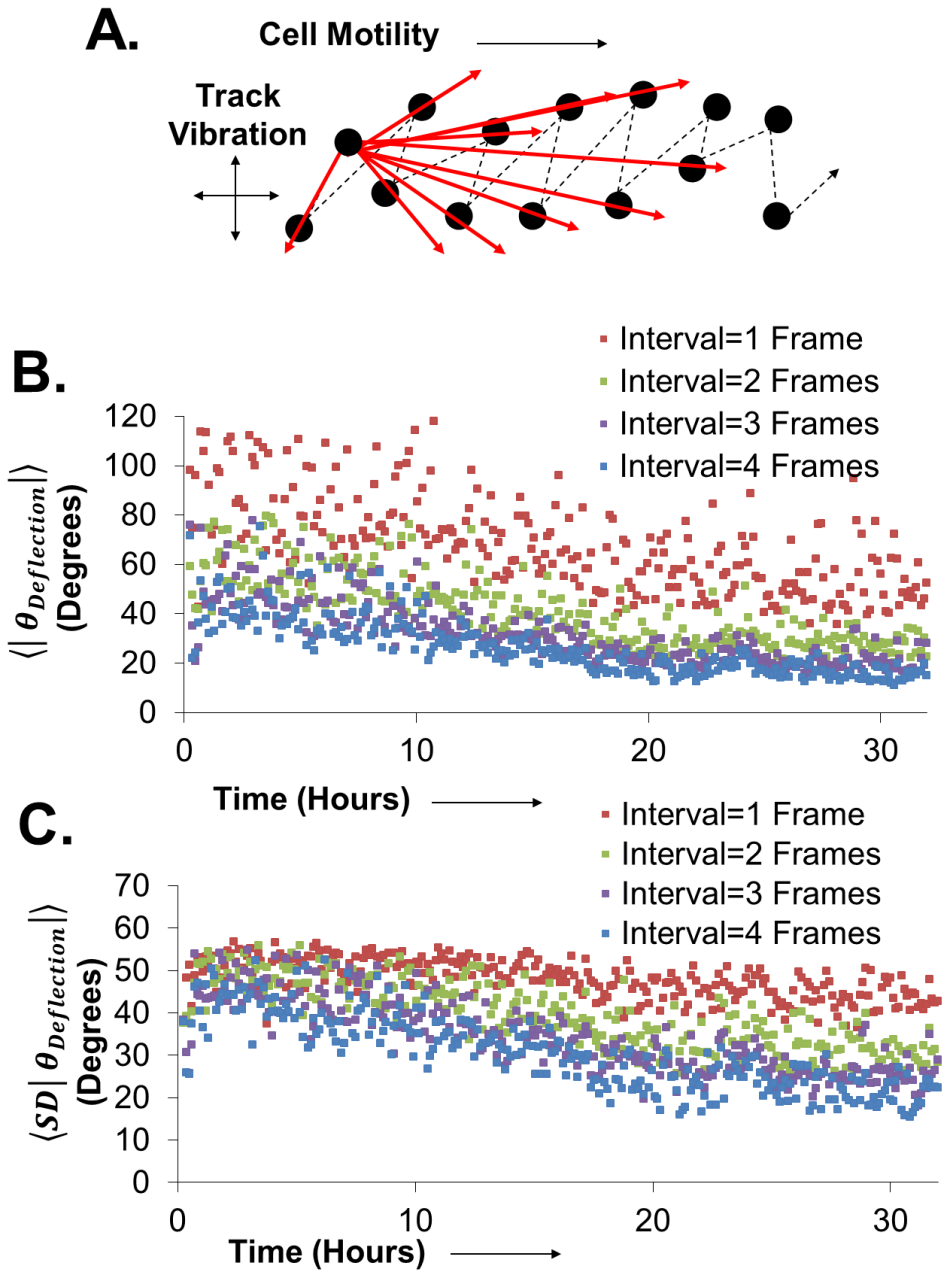
Overlapping Intervals Suppress Angular Noise in Cellular Migration. **A**) A schematic representation of a cellular track illustrates how increasing interval size results in the calculation of a cellular migration direction that is resistant to track vibration noise. **B**) The average absolute angle of deflection as a function of time for TGFβ treated HaCaT H2B mCherry cells for an interval size of one frame shows strong scattering of measurements. Upon increasing interval size, such scattering is suppressed. **C**) Such scattering can be also measured by measuring the standard deviation in the absolute angle of deflection, which can be suppressed in a similar manner by increasing interval size. Data represents greater than 1000 cells for each plot.

The Pathfinder software is unique from other software in that it calculates angular information about individual cells. However, such angular information requires that the determination of a cell's position be relatively noise free. When we closely examine the tracks of individual cells, we find that tracks exhibit slight vibration on the short timescale (7 minutes), such that a cell that migrates relatively straight does not display a perfectly straight track. As a result, we use overlapping intervals for our calculations of angular information in order to suppress the effects of such vibration on angular calculations. The schematic diagram in **Fig. 4A** illustrates how overlapping intervals aid in the reduction of noise in the calculation of cellular speed, direction, and persistence. Presented is the path of a single hypothetical cell that travels from positions 1 to 6. When calculating the trajectory of the movement from position 1 to 2, the resulting vector does not accurately represent the underlying trajectory of the cell over time. However, as the calculation is repeated in the same manner for a change in position from 1 to increasing successive positions, the resulting vectors quickly converge on the underlying trajectory of the cell. Each interval represents the cellular behavior in a video that has a frame rate that is the (acquisition frame rate)×(the interval size). For instance, if the interval size is 3 frames, then calculations are conducted on frames 1,4,7,10, and so forth. When successive intervals are combined, with a single frame shift from one interval to the next, the resulting data provides a time dependent parameter that has greatly suppressed noise.

**Fig. 4B and C** show the effect of increasing interval size on the time dependent average absolute angle of deflection trend and the average standard deviation of the absolute angle of deflection trend for HaCaT cells treated with TGFβ. With an interval size of 1 frame, both the average (**Fig. 4B**) and the error (**Fig. 4C**) of the angle deflection measurements are extremely noisy. With increasing interval size, such noise is suppressed, where an interval size of greater than 2 (corresponding to 14 minutes) does not yield significant additional suppression of noise. For all cellular experiments detailed in this investigation, an interval size of 3 frames was used. This method of overlapping intervals was applied to all measurements, with the exception of persistence time measurements.

### Measuring time dependent changes in migration persistence and speed

**Figure 5:**
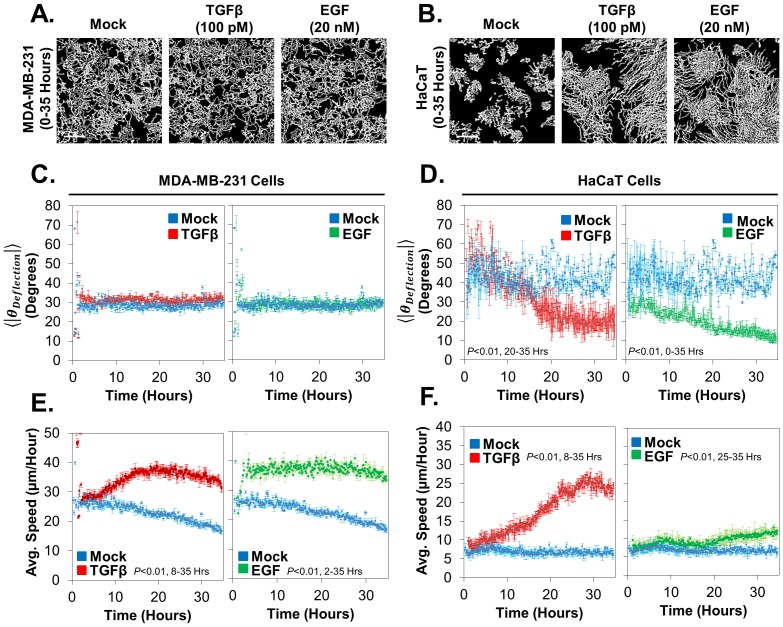
Measuring individual cellular behavior with speed and migration persistence reveals cell type and ligand specific cellular migration behavior. Cellular tracks of low density cells are displayed for treatments of either Mock, TGFβ or EGF for MDA-MB-231 cells (**A**) or HaCaT cells (**B**). Calibration bars represent 150 µm. **C**) Neither TGF-Beta nor EGF stimulation affects migration persistence in MDA-MB-231cells (top). In contrast, both treatments affect cellular speed, but with different induction kinetics (bottom). **D**) In HaCaT cells, both ligand treatments affect migration persistence and cellular speed (top and bottom, respectively). However, EGF stimulates migration persistence with earlier kinetics than that of TGFβ (top), and EGF is a poor stimulator of migration speed (bottom, right). Each condition represents greater than 1000 cells.

Upon mere qualitative assessment of cellular tracks, cellular behavior is difficult to deduce for large populations of cells. For example, when we examine MDA-MB-231 and HaCaT cell migration in response to either TGFβ or EGF by looking at the tracks of cells between 0 and 35 hours post ligand stimulation, MDA-MB-231 cells appear to not change behavior in response to ligand treatments and HaCaT cells appear to respond in the same manner upon TGFβ and EGF stimulation (**Fig. 5A and B**). However, MDA-MB-231 cells do in fact respond to ligand stimulations and HaCaT cells do in fact respond differently to TGFβ and EGF stimulation (**Movie S1 and S2**), which is elaborated upon below. Thus, only through rigorous quantitation can large populations be characterized for their cellular behavior.

Using our quantitative approach to measuring cellular migration, we were able to determine that either TGFβ or EGF causes MDA-MB-231 cells to migrate faster, but has almost no effect on how persistently cells migrate. Only stimulation with TGFβ causes a statistically significant (*p* = 0.003), but extremely small in magnitude, decrease in migration persistence, as evident by a slight elevation in the average absolute angle of deflection of cells (**Fig. 5C** **, top left**). Both TGFβ and EGF treatments yield an increase in the average speed of these cells, where EGF response is early (approximately 1 hour) and TGFβ response is late (approximately 10 hours) (**Fig. 5C** **, bottom**). In contrast to MDA-MB-231 cells, both speed and migration persistence are activated by TGFβ and EGF treatment in HaCaT cells, where the effects of EGF appear early (approximately 1 hour) and the effects of TGFβ appear late (approximately 10 hours) (**Fig. 5D**). Through our rigorous quantitation method, we conclude that motility promoting ligand stimulations can differ greatly from each other in terms of both the effect on cellular migration parameters and the kinetics of activation, both of which can be determined using the Pathfinder program. Taken together, this data illustrates the importance of time resolution in measuring cell migration responses to environmental stimuli, as well as the importance of measuring both the speed and the persistence of cells in order to characterize the full behavior of cell migration.

### Quantifying collective migration with nearest neighbor analyses of angular measurements

**Figure 6:**
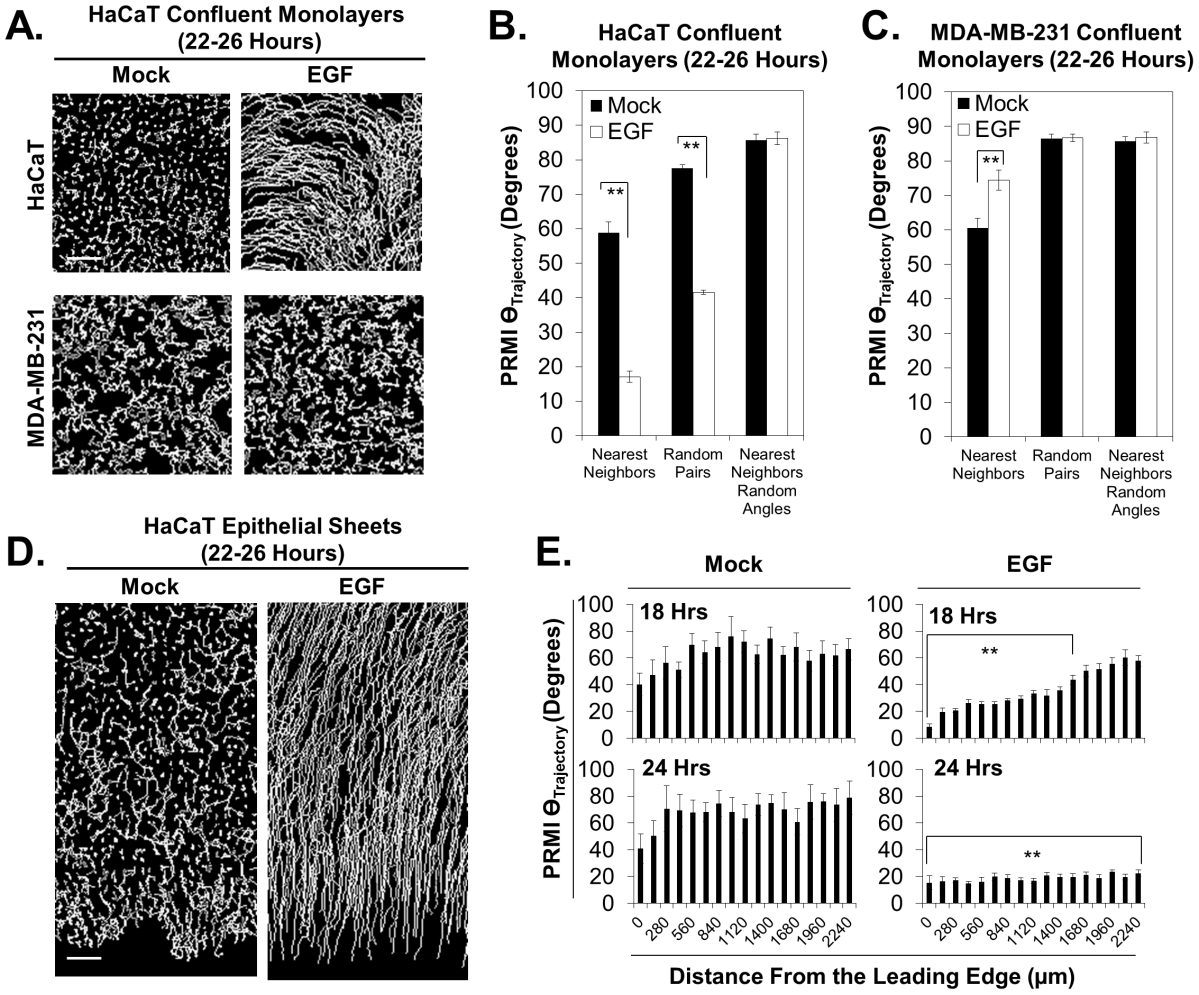
Angular measurements can be used to quantify collective migration behavior. **A**) Cellular tracks of confluent monolayers of HaCaT (top) and MDA-MB-231 cells (bottom) in the presence and absence of EGF stimulation. Calibration bar represents 150 µm. **B**) Confluent monolayers of HaCaT cells in the presence and absence of EGF stimulation were quantified for their collective migration behavior by calculating the average standard deviation of the angle of trajectory (also called the paired random migration index (PRMI Θ_Tajectory_) amongst nearest neighboring cells. Random pairing was used to determine whether the observed behavior was local or global amongst the population. **C**) The same quantification was conducted for MDA-MB-231 cells. **D**) Cellular tracks of epithelial sheets of HaCaT cells in the presence and absence of EGF stimulation. Calibration bar represents 150 µm. **E**) Inspection of the spatial distribution of collective migration behavior reveals that EGF stimulation elicits collective migration that propagates away from the leading edge. Double asterisks indicate a *p* value<0.01.

The increasing interest in the collective behavior of cells in the cell migration field prompted us to integrate the capability of characterizing collective migration into the Pathfinder software. One already implemented method for measuring collective migration involves the calculation of the size of collectively migrating streams of cells [33]. Although this method is certainly a valid way to measure the ‘collectiveness’ of cells within a population of cells, it requires a predetermined threshold to identify whether or not neighboring cells are part of a migrating stream (a 10 degree difference in migration direction or less is required to group cells together as a collectively migrating stream). In contrast, we used Pathfinder and a matrix based calculation to provide an alternative method for measuring how similar pairs of neighboring cells are in the directions that they migrate. This method does not require any predetermined thresholds as mention above. For each cell, Pathfinder reports the angle of trajectory, or migration direction. For this parameter, each cell is assigned an angle that ranges from 0 to 359 degrees relative to a well-defined set of axes in the field of view. **Fig. 2B** **, right**, illustrates the orientation of these axes in the field of view, and provides an example of a cell that is migrating in the direction of 45 degrees. In a similar manner to the case of angle of deflection, each cell gets such an assignment for each time, providing time resolution of cellular direction. In order to demonstrate how the angle of trajectory calculation can be used to characterize collective migration, we examined HaCaT and MDA-MB-231 cells in confluent monolayers in response to EGF stimulation. Manual inspection of the cellular tracks of these monolayers suggests that neighboring cells migrate in a similar direction in a ligand dependent manner in HaCaT cells (**Fig. 6A** **, top**). This behavior is not qualitatively observed for MDA-MB-231 cells (**Fig. 6A** **, bottom**). Since collective migration is defined as the ability of cells to adopt a common migration direction, we quantified the average standard deviation of the angle of trajectory, also referred to as the “paired random migration index” (PRMI Θ_Trajectory_), amongst pairs of nearest neighboring cells at 22–26 hours post ligand stimulation. An increase in this quantity indicates that nearest neighboring cells are migrating in increasingly different directions, meaning collective migration is decreasing. We excluded pairs of neighbors in which one cell migrates with a direction of 0–90 degrees and another cell migrates in a direction of 270–360 degrees, as these pairs would have a falsely high standard deviation due to the discontinuous transition between 360 degrees and 1 degrees. In agreement with our qualitative observations of cellular tracks, nearest neighboring HaCaT cells display a lower PRMI Θ_Trajectory_ in response to EGF stimulation (**Fig. 6B**). We compared nearest neighbor behavior to random pairing behavior in order to identify when shared behavior of cells is global versus local. If random pairing does not change the magnitude of the PRMI Θ_Trajectory_, then the shared behavior is entirely global. In contrast, when such magnitudes are affected by random pairing, the phenomenon is local. Upon random pairing of HaCaT cells, a similar trend is observed for the PRMI Θ_Trajectory_, but the magnitudes increase for both mock treatment and EGF treatment. Thus, EGF stimulation of HaCaT cells activates local collective migration, which diminishes with increasing distance between neighbors. As a control, we repeated our nearest neighbor calculations after substitution of random angles of trajectory (nearest neighbors random angles) in order to determine that the maximum value for the PRMI Θ_Trajectory_ is approximately 85 degrees in both the presence and absence of EGF stimulation. This maximum value is greater than that of the PRMI Θ_Trajectory_ in the absence of ligand stimulation, revealing that HaCaT cells do display a small degree of collective migration, which we were not able to detect upon manual qualitative inspection of time-lapse videos. Using the same technique on MDA-MB-231 cells, we found that these cells display a statistically significant, but low magnitude, increase in the PRMI Θ_Trajectory_ amongst nearest neighbors in response to EGF, suggesting that ligand stimulation of these cells causes neighboring cells to exhibit slight repulsion, and migrate more in opposing directions upon EGF stimulation (**Fig. 6C**). Random pairing of MDA-MB-231 cells led to an increase in the magnitude of the PRMI Θ_Trajectory_, revealing that the collective migration of these cells is entirely a local phenomenon. Substitution of random angles of trajectories into data sets revealed a similar maximum value for the PRMI Θ_Trajectory_, which was approximately 85 degrees. Whether or not the apparent collective migration behavior of neighboring MDA-MB-231 cells in the absence of ligand stimulation constitutes collective migration according to the accepted definition will require further investigation into the requirement of cellular junctions in this process. However, it is worth noting that the neighboring cells do in fact have physical contact with each other (**Fig. S4**), albeit for short timescales (data not shown).

### Using nearest neighbor analyses of angular measurements to characterize collective migration in epithelial sheets

In order to determine the versatility of our method of collective migration quantification, we repeated our experiments on HaCaT cells using EGF stimulation, but under the condition in which cells were arranged into epithelial sheets (**Fig. 6D** **and Movie S3**). We asked whether or not ligand stimulation instantaneously affects all cells equally in an epithelial sheet, and found that the collective behavior response to EGF stimulation propagates away from the leading edge of an epithelial sheet overtime as indicated by propagation of decreasing PRMI Θ_Trajectory_ away from the leading edge of an epithelial sheet (**Fig. 6E**). Thus, our technique of quantitatively characterizing collective migration has proven useful to determine the spatial collective migration properties throughout a population of cells.

## Discussion

### The pathfinder software provides novel useful tools for studying individual and collective cell migration

Our Pathfinder software is the first high throughput automated cell migration software to conduct measurements of instantaneous angular parameters for the quantification of cell migration behavior. Our parameter of the angle of deflection has proven useful for characterizing the migration persistence of cells in two dimensional fluorescence microscopy experiments. In addition to helping researchers investigate the molecular mechanisms behind migration persistence, this parameter can be used to identify the differential temporal responses of cells to distinct ligand stimulations, and can provide insight into the molecular mechanisms behind each ligand response. Furthermore, the available cell migration software has been entirely devoid of tools that can be used to readily quantify collective migration. As a result, investigations of collective migration have been more appropriately described as qualitative rather than quantitative. The Pathfinder software provides the first high throughput tool to quantitatively characterize collective cell migration using the angle of trajectory parameter, which can be used to determine the “Paired Random Migration Index” (PRMI Θ_Trajectory_) amongst nearest neighbors. This tool is likely to propel the collective migration field forward, as it can provide a means for accurately measuring the degree to which cells are migrating collectively, with both spatial and temporal resolution. Our method of measuring collective migration was able to reveal hidden collective cellular behavior that cannot be easily detected qualitatively from cellular tracks, such as the local collective migration behavior amongst pairs of MDA-MB-231 cells, and the ability of EGF stimulation to suppress this behavior. In conclusion, our Pathfinder software provides novel techniques for characterizing cellular migration in a high throughput platform, which are likely to aid the cell migration field in its investigation of the molecular mechanisms behind individual and collective migration.

## Supporting Information

Data S1 **Pathfinder Program.** The Pathfinder program is an executable JAVA program that requires installation of JAVA Runtime Environment on a Windows operating system.
(JAR)

Data S2 **A sample output from Pathfinder.** A sheet of HaCaT H2B-mCherry cells were analyzed for individual cell migration from a microscopy video between 22 and 25.5 hours post 100 nM EGF stimulation. Frames were acquired every 7 minutes. Analysis was conducted with a frame binning of 3 frames (21 minutes).
(XLSX)

Data S3 **A MATLAB script to convert mean squared displacement (MSD) versus time data from a Pathfinder output into persistence time.** This MATLAB script requires user input of MSD and time data for each cell and converts this information into persistence time.
(M) Click here for additional data file.

Figure S1 **Parameter descriptions for the Pathfinder program GUI.** User input parameters are: Cell Outer Radius, Cell Minimum Radius (Cutoff), Percentage of Pixels with Nuclear Signal (Percentile), How Far an Average Cell is Tolerated to Migrate From Frame to Frame (Disp./Frame), How Many Frames to Bin for Calculations (Frame Binning), Minimum Tack Length (Min. Trajectory Length), Number of Parallel Threads (Threads), Folder Path for Folder with Videos (AVI Folder).
(TIF)

Figure S2 **A description of output calculations from the Pathfinder program.** Each cell receives a cellular ID number (1), for each frame (2). In each frame a cell is assigned an X (3) and Y (4) coordinate, a displacement from the last frame in pixels (5), an angle of trajectory (6), an angle of deflection (7) and a mean squared displacement (8). Mean squared displacements can be used to calculate the persistence time for a cell. For the population of cells, Pathfinder reports the frame (9) dependent change in the average displacement (10), the average angle of trajectory (11), the percentage of cells turning greater than 90 degrees (12), and the average absolute angle of deflection (13). Additionally, Pathfinder reports a binned histogram of percent of cells versus the possible migration directions from 0 to 359 degrees (14 and 15). Lastly, the number of cellular tracks is reported (16).
(TIF)

Figure S3 **Wild type MDA-MB-231 cells and MDA-MB-231 H2B-mCherry cells migrate with similar speeds in the presence and absence of EGF stimulation.** Brightfield microscopy videos of mock and EGF treated wild type (WT) MDA-MB-231 cells were manually measured for position over the course of a 10 frame interval (7 minutes/frame) after 24 hours ligand or mock stimulation and the average speed of cells was calculated with a frame binning of 3. The same analysis was done on parallel videos of MDA-MB-231 using pathfinder, which yielded similar results for WT and labeled cells in the speed of migration in the presence and absence of EGF. 50 cells were used for this comparison for each condition.
(TIF)

Figure S4 **MDA-MB-231 cells maintain physical contact with their nearest neighboring cell.** Brightfield microscopy of EGF treated MDA-MB-231 cells reveals that nearest neighboring cells have physical contact with each other.
(TIF)

Movie S1 **MDA-MB-231 cells at low density upon either mock, TGFβ, or EGF treatment.** MDA-MB-231 cells with an H2B-mCherry nuclear marker were observed by time-lapse microscopy using the mCherry fluorescence channel. Each frame represents 7 minutes.
(AVI)

Movie S2 **HaCaT cells at low density upon either mock, TGFβ, or EGF treatment.** HaCaT cells with an H2B-mCherry nuclear marker were observed by time-lapse microscopy using the mCherry fluorescence channel. Each frame represents 7 minutes.
(AVI)

Movie S3 **Epithelial sheets of HaCaT cells upon either mock or EGF treatment.** HaCaT cells with an H2B-mCherry nuclear marker were assembled into epithelial sheets and observed by time-lapse microscopy using the mCherry fluorescence channel. Each frame represents 7 minutes.
(AVI)

## References

[1] Chambers AF, Groom AC, MacDonald IC (2002) Dissemination and growth of cancer cells in metastatic sites. Nat Rev Cancer 2: 563–572. 12154349 10.1038/nrc865

[2] Friedl P, Wolf K (2003) Tumour-cell invasion and migration: diversity and escape mechanisms. Nat Rev Cancer 3: 362–374. 12724734 10.1038/nrc1075

[3] Keller R (2005) Cell migration during gastrulation. Curr Opin Cell Biol 17: 533–541. 16099638 10.1016/j.ceb.2005.08.006

[4] Locascio A, Nieto MA (2001) Cell movements during vertebrate development: integrated tissue behaviour versus individual cell migration. Curr Opin Genet Dev 11: 464–469. 11448634 10.1016/s0959-437x(00)00218-5

[5] Montell DJ (2003) Border-cell migration: the race is on. Nat Rev Mol Cell Biol 4: 13–24. 12511865 10.1038/nrm1006

[6] Wang W, Goswami S, Lapidus K, Wells AL, Wyckoff JB, et al (2004) Identification and testing of a gene expression signature of invasive carcinoma cells within primary mammary tumors. Cancer Res 64: 8585–8594. 15574765 10.1158/0008-5472.CAN-04-1136

[7] Wang W, Goswami S, Sahai E, Wyckoff JB, Segall JE, et al (2005) Tumor cells caught in the act of invading: their strategy for enhanced cell motility. Trends Cell Biol 15: 138–145. 15752977 10.1016/j.tcb.2005.01.003

[8] Abercrombie M (1979) Contact inhibition and malignancy. Nature 281: 259–262. 551275 10.1038/281259a0

[9] Abercrombie M, Heaysman JE (1953) Observations on the social behaviour of cells in tissue culture. I. Speed of movement of chick heart fibroblasts in relation to their mutual contacts. Exp Cell Res 5: 111–131. 13083622 10.1016/0014-4827(53)90098-6

[10] Abercrombie M, Heaysman JE (1954) Invasiveness of sarcoma cells. Nature 174: 697–698. 13213985 10.1038/174697a0

[11] Horwitz R, Webb D (2003) Cell migration. Curr Biol 13: R756–759. 14521851 10.1016/j.cub.2003.09.014

[12] Lauffenburger DA, Horwitz AF (1996) Cell migration: a physically integrated molecular process. Cell 84: 359–369. 8608589 10.1016/s0092-8674(00)81280-5

[13] Van Haastert PJ, Devreotes PN (2004) Chemotaxis: signalling the way forward. Nat Rev Mol Cell Biol 5: 626–634. 15366706 10.1038/nrm1435

[14] Rorth P (2009) Collective cell migration. Annu Rev Cell Dev Biol 25: 407–429. 19575657 10.1146/annurev.cellbio.042308.113231

[15] Rorth P (2007) Collective guidance of collective cell migration. Trends Cell Biol 17: 575–579. 17996447 10.1016/j.tcb.2007.09.007

[16] Bronner-Fraser M (1993) Neural crest cell migration in the developing embryo. Trends Cell Biol 3: 392–397. 14731657 10.1016/0962-8924(93)90089-j

[17] Lois C, Garcia-Verdugo JM, Alvarez-Buylla A (1996) Chain migration of neuronal precursors. Science 271: 978–981. 8584933 10.1126/science.271.5251.978

[18] Martin P (1997) Wound healing–aiming for perfect skin regeneration. Science 276: 75–81. 9082989 10.1126/science.276.5309.75

[19] Murase S, Horwitz AF (2004) Directions in cell migration along the rostral migratory stream: the pathway for migration in the brain. Curr Top Dev Biol 61: 135–152. 15350400 10.1016/S0070-2153(04)61006-4

[20] Rorth P (2002) Initiating and guiding migration: lessons from border cells. Trends Cell Biol 12: 325–331. 12185849 10.1016/s0962-8924(02)02311-5

[21] Xia Y, Karin M (2004) The control of cell motility and epithelial morphogenesis by Jun kinases. Trends Cell Biol 14: 94–101. 15102441 10.1016/j.tcb.2003.12.005

[22] Zhang L, Wang W, Hayashi Y, Jester JV, Birk DE, et al (2003) A role for MEK kinase 1 in TGF-beta/activin-induced epithelium movement and embryonic eyelid closure. Embo J 22: 4443–4454. 12941696 10.1093/emboj/cdg440 PMC202382

[23] Abercrombie M, Heaysman JE (1954) Observations on the social behaviour of cells in tissue culture. II. Monolayering of fibroblasts. Exp Cell Res 6: 293–306. 13173482 10.1016/0014-4827(54)90176-7

[24] Ridley AJ, Schwartz MA, Burridge K, Firtel RA, Ginsberg MH, et al (2003) Cell migration: integrating signals from front to back. Science 302: 1704–1709. 14657486 10.1126/science.1092053

[25] Fischer RS, Gardel M, Ma X, Adelstein RS, Waterman CM (2009) Local cortical tension by myosin II guides 3D endothelial cell branching. Curr Biol 19: 260–265. 19185493 10.1016/j.cub.2008.12.045 PMC2777763

[26] Friedl P, Noble PB, Walton PA, Laird DW, Chauvin PJ, et al (1995) Migration of coordinated cell clusters in mesenchymal and epithelial cancer explants in vitro. Cancer Res 55: 4557–4560. 7553628

[27] Ghabrial A, Luschnig S, Metzstein MM, Krasnow MA (2003) Branching morphogenesis of the Drosophila tracheal system. Annu Rev Cell Dev Biol 19: 623–647. 14570584 10.1146/annurev.cellbio.19.031403.160043

[28] Khalil AA, Friedl P (2010) Determinants of leader cells in collective cell migration. Integr Biol (Camb) 2: 568–574. 20886167 10.1039/c0ib00052c

[29] Prasad M, Montell DJ (2007) Cellular and molecular mechanisms of border cell migration analyzed using time-lapse live-cell imaging. Dev Cell 12: 997–1005. 17543870 10.1016/j.devcel.2007.03.021

[30] Theveneau E, Marchant L, Kuriyama S, Gull M, Moepps B, et al (2010) Collective chemotaxis requires contact-dependent cell polarity. Dev Cell 19: 39–53. 20643349 10.1016/j.devcel.2010.06.012 PMC2913244

[31] Vitorino P, Hammer M, Kim J, Meyer T (2011) A steering model of endothelial sheet migration recapitulates monolayer integrity and directed collective migration. Mol Cell Biol 31: 342–350. 20974808 10.1128/MCB.00800-10 PMC3019974

[32] Vitorino P, Meyer T (2008) Modular control of endothelial sheet migration. Genes Dev 22: 3268–3281. 19056882 10.1101/gad.1725808 PMC2600767

[33] Slater B, Londono C, McGuigan AP (2013) An algorithm to quantify correlated collective cell migration behavior. Biotechniques 54: 87–92. 23384179 10.2144/000113990

[34] Chen Y, Dodd SJ, Tangrea MA, Emmert-Buck MR, Koretsky AP (2013) Measuring collective cell movement and extracellular matrix interactions using magnetic resonance imaging. Sci Rep 3: 1879. 23698816 10.1038/srep01879 PMC3662010

[35] Rosello C, Ballet P, Planus E, Tracqui P (2004) Model driven quantification of individual and collective cell migration. Acta Biotheor 52: 343–363. 15520538 10.1023/B:ACBI.0000046602.58202.5e

[36] dos Remedios CG, Chhabra D, Kekic M, Dedova IV, Tsubakihara M, et al (2003) Actin binding proteins: regulation of cytoskeletal microfilaments. Physiol Rev 83: 433–473. 12663865 10.1152/physrev.00026.2002

[37] Cory GO, Ridley AJ (2002) Cell motility: braking WAVEs. Nature 418: 732–733. 12181548 10.1038/418732a

[38] Etienne-Manneville S, Hall A (2002) Rho GTPases in cell biology. Nature 420: 629–635. 12478284 10.1038/nature01148

[39] Itoh RE, Kurokawa K, Ohba Y, Yoshizaki H, Mochizuki N, et al (2002) Activation of rac and cdc42 video imaged by fluorescent resonance energy transfer-based single-molecule probes in the membrane of living cells. Mol Cell Biol 22: 6582–6591. 12192056 10.1128/MCB.22.18.6582-6591.2002 PMC135619

[40] Mogilner A, Oster G (1996) Cell motility driven by actin polymerization. Biophys J 71: 3030–3045. 8968574 10.1016/S0006-3495(96)79496-1 PMC1233792

[41] Ridley AJ, Hall A (1992) The small GTP-binding protein rho regulates the assembly of focal adhesions and actin stress fibers in response to growth factors. Cell 70: 389–399. 1643657 10.1016/0092-8674(92)90163-7

[42] Worthylake RA, Burridge K (2003) RhoA and ROCK promote migration by limiting membrane protrusions. J Biol Chem 278: 13578–13584. 12574166 10.1074/jbc.M211584200

[43] Pollard TD, Borisy GG (2003) Cellular motility driven by assembly and disassembly of actin filaments. Cell 112: 453–465. 12600310 10.1016/s0092-8674(03)00120-x

[44] Welch MD, Mullins RD (2002) Cellular control of actin nucleation. Annu Rev Cell Dev Biol 18: 247–288. 12142287 10.1146/annurev.cellbio.18.040202.112133

[45] Rafelski SM, Theriot JA (2004) Crawling toward a unified model of cell mobility: spatial and temporal regulation of actin dynamics. Annu Rev Biochem 73: 209–239. 15189141 10.1146/annurev.biochem.73.011303.073844

[46] Condeelis JS, Wyckoff JB, Bailly M, Pestell R, Lawrence D, et al (2001) Lamellipodia in invasion. Semin Cancer Biol 11: 119–128. 11322831 10.1006/scbi.2000.0363

[47] Hussain NK, Jenna S, Glogauer M, Quinn CC, Wasiak S, et al (2001) Endocytic protein intersectin-l regulates actin assembly via Cdc42 and N-WASP. Nat Cell Biol 3: 927–932. 11584276 10.1038/ncb1001-927

[48] Soderling SH, Binns KL, Wayman GA, Davee SM, Ong SH, et al (2002) The WRP component of the WAVE-1 complex attenuates Rac-mediated signalling. Nat Cell Biol 4: 970–975. 12447388 10.1038/ncb886

[49] Nobes CD, Hall A (1995) Rho, rac, and cdc42 GTPases regulate the assembly of multimolecular focal complexes associated with actin stress fibers, lamellipodia, and filopodia. Cell 81: 53–62. 7536630 10.1016/0092-8674(95)90370-4

[50] Kurokawa K, Itoh RE, Yoshizaki H, Nakamura YO, Matsuda M (2004) Coactivation of Rac1 and Cdc42 at lamellipodia and membrane ruffles induced by epidermal growth factor. Mol Biol Cell 15: 1003–1010. 14699061 10.1091/mbc.E03-08-0609 PMC363057

[51] Moissoglu K, Schwartz MA (2006) Integrin signalling in directed cell migration. Biol Cell 98: 547–555. 16907663 10.1042/BC20060025

[52] Galbraith CG, Yamada KM, Galbraith JA (2007) Polymerizing actin fibers position integrins primed to probe for adhesion sites. Science 315: 992–995. 17303755 10.1126/science.1137904

[53] Burridge K, Chrzanowska-Wodnicka M, Zhong C (1997) Focal adhesion assembly. Trends Cell Biol 7: 342–347. 17708978 10.1016/S0962-8924(97)01127-6

[54] Webb DJ, Parsons JT, Horwitz AF (2002) Adhesion assembly, disassembly and turnover in migrating cells – over and over and over again. Nat Cell Biol 4: E97–100. 11944043 10.1038/ncb0402-e97

[55] Burridge K, Chrzanowska-Wodnicka M (1996) Focal adhesions, contractility, and signaling. Annu Rev Cell Dev Biol 12: 463–518. 8970735 10.1146/annurev.cellbio.12.1.463

[56] Ren XD, Kiosses WB, Sieg DJ, Otey CA, Schlaepfer DD, et al (2000) Focal adhesion kinase suppresses Rho activity to promote focal adhesion turnover. J Cell Sci 113 Pt 20: 3673–3678. 11017882 10.1242/jcs.113.20.3673

[57] Webb DJ, Donais K, Whitmore LA, Thomas SM, Turner CE, et al (2004) FAK-Src signalling through paxillin, ERK and MLCK regulates adhesion disassembly. Nat Cell Biol 6: 154–161. 14743221 10.1038/ncb1094

[58] Burridge K, Turner CE, Romer LH (1992) Tyrosine phosphorylation of paxillin and pp125FAK accompanies cell adhesion to extracellular matrix: a role in cytoskeletal assembly. J Cell Biol 119: 893–903. 1385444 10.1083/jcb.119.4.893 PMC2289706

[59] Cuevas BD, Abell AN, Witowsky JA, Yujiri T, Johnson NL, et al (2003) MEKK1 regulates calpain-dependent proteolysis of focal adhesion proteins for rear-end detachment of migrating fibroblasts. Embo J 22: 3346–3355. 12839996 10.1093/emboj/cdg322 PMC165646

[60] Lee J, Ishihara A, Oxford G, Johnson B, Jacobson K (1999) Regulation of cell movement is mediated by stretch-activated calcium channels. Nature 400: 382–386. 10432119 10.1038/22578

[61] Ware MF, Wells A, Lauffenburger DA (1998) Epidermal growth factor alters fibroblast migration speed and directional persistence reciprocally and in a matrix-dependent manner. J Cell Sci 111 Pt 16: 2423–2432. 9683636 10.1242/jcs.111.16.2423

[62] Xie H, Pallero MA, Gupta K, Chang P, Ware MF, et al (1998) EGF receptor regulation of cell motility: EGF induces disassembly of focal adhesions independently of the motility-associated PLCgamma signaling pathway. J Cell Sci 111 Pt 5: 615–624. 9454735 10.1242/jcs.111.5.615

[63] Gail MH, Boone CW (1970) The locomotion of mouse fibroblasts in tissue culture. Biophys J 10: 980–993. 5531614 10.1016/S0006-3495(70)86347-0 PMC1367974

[64] Hall A (1994) Small GTP-binding proteins and the regulation of the actin cytoskeleton. Annu Rev Cell Biol 10: 31–54. 7888179 10.1146/annurev.cb.10.110194.000335

[65] Houk AR, Jilkine A, Mejean CO, Boltyanskiy R, Dufresne ER, et al (2012) Membrane tension maintains cell polarity by confining signals to the leading edge during neutrophil migration. Cell 148: 175–188. 22265410 10.1016/j.cell.2011.10.050 PMC3308728

[66] Mitra SK, Hanson DA, Schlaepfer DD (2005) Focal adhesion kinase: in command and control of cell motility. Nat Rev Mol Cell Biol 6: 56–68. 15688067 10.1038/nrm1549

[67] Small JV, Kaverina I (2003) Microtubules meet substrate adhesions to arrange cell polarity. Curr Opin Cell Biol 15: 40–47. 12517702 10.1016/s0955-0674(02)00008-x

[68] Srinivasan S, Wang F, Glavas S, Ott A, Hofmann F, et al (2003) Rac and Cdc42 play distinct roles in regulating PI(3,4,5)P3 and polarity during neutrophil chemotaxis. J Cell Biol 160: 375–385. 12551955 10.1083/jcb.200208179 PMC2172671

[69] Weber GF, Bjerke MA, DeSimone DW (2012) A mechanoresponsive cadherin-keratin complex directs polarized protrusive behavior and collective cell migration. Dev Cell 22: 104–115. 22169071 10.1016/j.devcel.2011.10.013 PMC3264825

[70] Zamir E, Geiger B (2001) Molecular complexity and dynamics of cell-matrix adhesions. J Cell Sci 114: 3583–3590. 11707510 10.1242/jcs.114.20.3583

[71] Bray K, Brakebusch C, Vargo-Gogola T (2011) The Rho GTPase Cdc42 is required for primary mammary epithelial cell morphogenesis in vitro. Small GTPases 2: 247–258. 22292127 10.4161/sgtp.2.5.18163 PMC3265815

[72] Osmani N, Peglion F, Chavrier P, Etienne-Manneville S (2010) Cdc42 localization and cell polarity depend on membrane traffic. J Cell Biol 191: 1261–1269. 21173111 10.1083/jcb.201003091 PMC3010071

[73] Sieg DJ, Hauck CR, Ilic D, Klingbeil CK, Schaefer E, et al (2000) FAK integrates growth-factor and integrin signals to promote cell migration. Nat Cell Biol 2: 249–256. 10806474 10.1038/35010517

[74] Sieg DJ, Hauck CR, Schlaepfer DD (1999) Required role of focal adhesion kinase (FAK) for integrin-stimulated cell migration. J Cell Sci 112 Pt 16: 2677–2691. 10413676 10.1242/jcs.112.16.2677

[75] Cukierman E, Pankov R, Stevens DR, Yamada KM (2001) Taking cell-matrix adhesions to the third dimension. Science 294: 1708–1712. 11721053 10.1126/science.1064829

[76] Fraley SI, Feng Y, Krishnamurthy R, Kim DH, Celedon A, et al (2010) A distinctive role for focal adhesion proteins in three-dimensional cell motility. Nat Cell Biol 12: 598–604. 20473295 10.1038/ncb2062 PMC3116660

[77] Tang H, Li A, Bi J, Veltman DM, Zech T, et al (2013) Loss of Scar/WAVE complex promotes N-WASP- and FAK-dependent invasion. Curr Biol 23: 107–117. 23273897 10.1016/j.cub.2012.11.059

[78] Kostic A, Lynch CD, Sheetz MP (2009) Differential matrix rigidity response in breast cancer cell lines correlates with the tissue tropism. PLoS One 4: e6361. 19626122 10.1371/journal.pone.0006361 PMC2709918

[79] Boldajipour B, Mahabaleshwar H, Kardash E, Reichman-Fried M, Blaser H, et al (2008) Control of chemokine-guided cell migration by ligand sequestration. Cell 132: 463–473. 18267076 10.1016/j.cell.2007.12.034

[80] Tang Q, Adams JY, Tooley AJ, Bi M, Fife BT, et al (2006) Visualizing regulatory T cell control of autoimmune responses in nonobese diabetic mice. Nat Immunol 7: 83–92. 16311599 10.1038/ni1289 PMC3057888

[81] Wolf K, Wu YI, Liu Y, Geiger J, Tam E, et al (2007) Multi-step pericellular proteolysis controls the transition from individual to collective cancer cell invasion. Nat Cell Biol 9: 893–904. 17618273 10.1038/ncb1616

[82] Harms BD, Bassi GM, Horwitz AR, Lauffenburger DA (2005) Directional persistence of EGF-induced cell migration is associated with stabilization of lamellipodial protrusions. Biophys J 88: 1479–1488. 15713602 10.1529/biophysj.104.047365 PMC1305149

[83] Huth J, Buchholz M, Kraus JM, Molhave K, Gradinaru C (2011) TimeLapseAnalyzer: multi-target analysis for live-cell imaging and time-lapse microscopy. Comput Methods Programs Biomed 104: 227–234. 21705106 10.1016/j.cmpb.2011.06.002

[84] Huth J, Buchholz M, Kraus JM, Schmucker M, von Wichert G (2010) Significantly improved precision of cell migration analysis in time-lapse video microscopy through use of a fully automated tracking system. BMC Cell Biol 11: 24. 20377897 10.1186/1471-2121-11-24 PMC2858025

[85] Joslin EJ, Opresko LK, Wells A, Wiley HS, Lauffenburger DA (2007) EGF-receptor-mediated mammary epithelial cell migration is driven by sustained ERK signaling from autocrine stimulation. J Cell Sci 120: 3688–3699. 17895366 10.1242/jcs.010488

[86] Platek A, Mettlen M, Camby I, Kiss R, Amyere M, et al (2004) v-Src accelerates spontaneous motility via phosphoinositide 3-kinase, phospholipase C and phospholipase D, but abrogates chemotaxis in Rat-1 and MDCK cells. J Cell Sci 117: 4849–4861. 15340010 10.1242/jcs.01359

[87] Sawyer C, Sturge J, Bennett DC, O'Hare MJ, Allen WE, et al (2003) Regulation of breast cancer cell chemotaxis by the phosphoinositide 3-kinase p110delta. Cancer Res 63: 1667–1675. 12670921

[88] Sturge J, Hamelin J, Jones GE (2002) N-WASP activation by a beta1-integrin-dependent mechanism supports PI3K-independent chemotaxis stimulated by urokinase-type plasminogen activator. J Cell Sci 115: 699–711. 11865026 10.1242/jcs.115.4.699

[89] Sbalzarini IF, Koumoutsakos P (2005) Feature point tracking and trajectory analysis for video imaging in cell biology. J Struct Biol 151: 182–195. 16043363 10.1016/j.jsb.2005.06.002

