# Supplementary figures and images for: The Development of a Novel High Throughput Computational Tool for Studying Individual and Collective Cellular Migration
Source: PLoS One. 2013 Dec 27;8(12):e82444. doi: 10.1371/journal.pone.0082444 (PMC3873918; doi:10.1371/journal.pone.0082444)

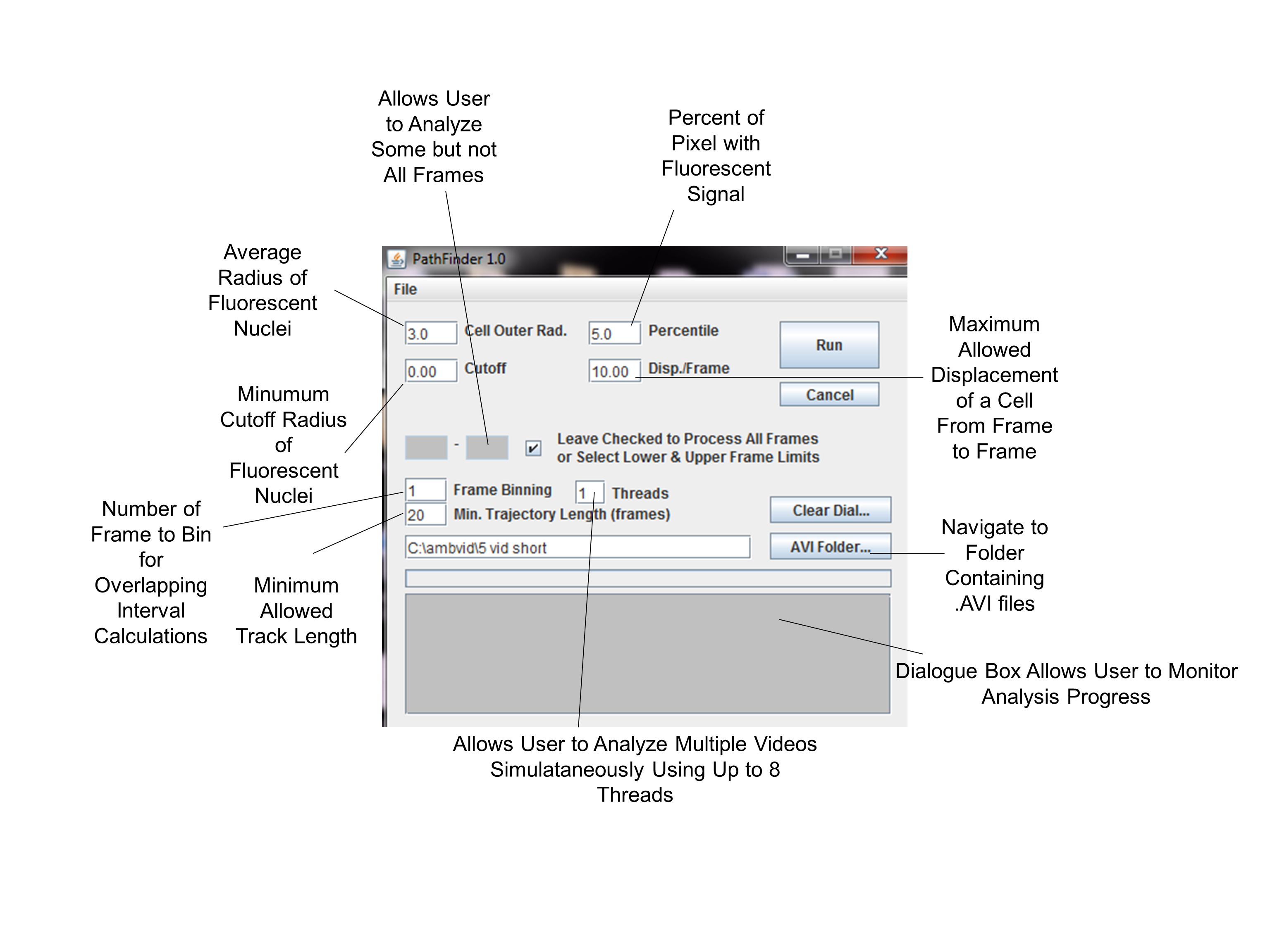

Supplement: Figure S1 — Parameter descriptions for the Pathfinder program GUI. User input parameters are: Cell Outer Radius, Cell Minimum Radius (Cutoff), Percentage of Pixels with Nuclear Signal (Percentile), How Far an Average Cell is Tolerated to Migrate From Frame to Frame (Disp./Frame), How Many Frames to Bin for Calculations (Frame Binning), Minimum Tack Length (Min. Trajectory Length), Number of Parallel Threads (Threads), Folder Path for Folder with Videos (AVI Folder). (TIF) [file pone.0082444.s004.tif]

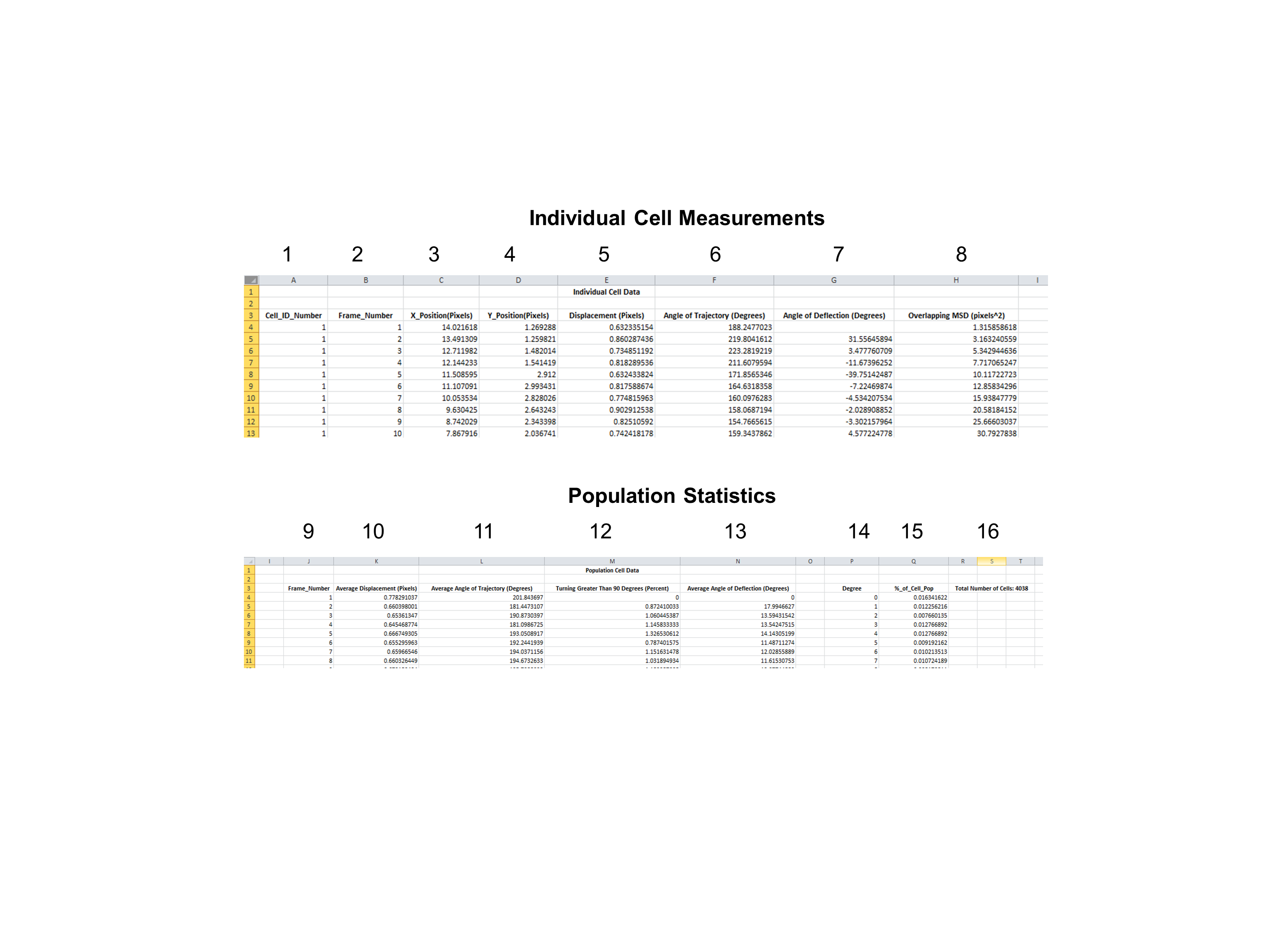

Supplement: Figure S2 — A description of output calculations from the Pathfinder program. Each cell receives a cellular ID number (1), for each frame (2). In each frame a cell is assigned an X (3) and Y (4) coordinate, a displacement from the last frame in pixels (5), an angle of trajectory (6), an angle of deflection (7) and a mean squared displacement (8). Mean squared displacements can be used to calculate the persistence time for a cell. For the population of cells, Pathfinder reports the frame (9) dependent change in the average displacement (10), the average angle of trajectory (11), the percentage of cells turning greater than 90 degrees (12), and the average absolute angle of deflection (13). Additionally, Pathfinder reports a binned histogram of percent of cells versus the possible migration directions from 0 to 359 degrees (14 and 15). Lastly, the number of cellular tracks is reported (16). (TIF) [file pone.0082444.s005.tif]

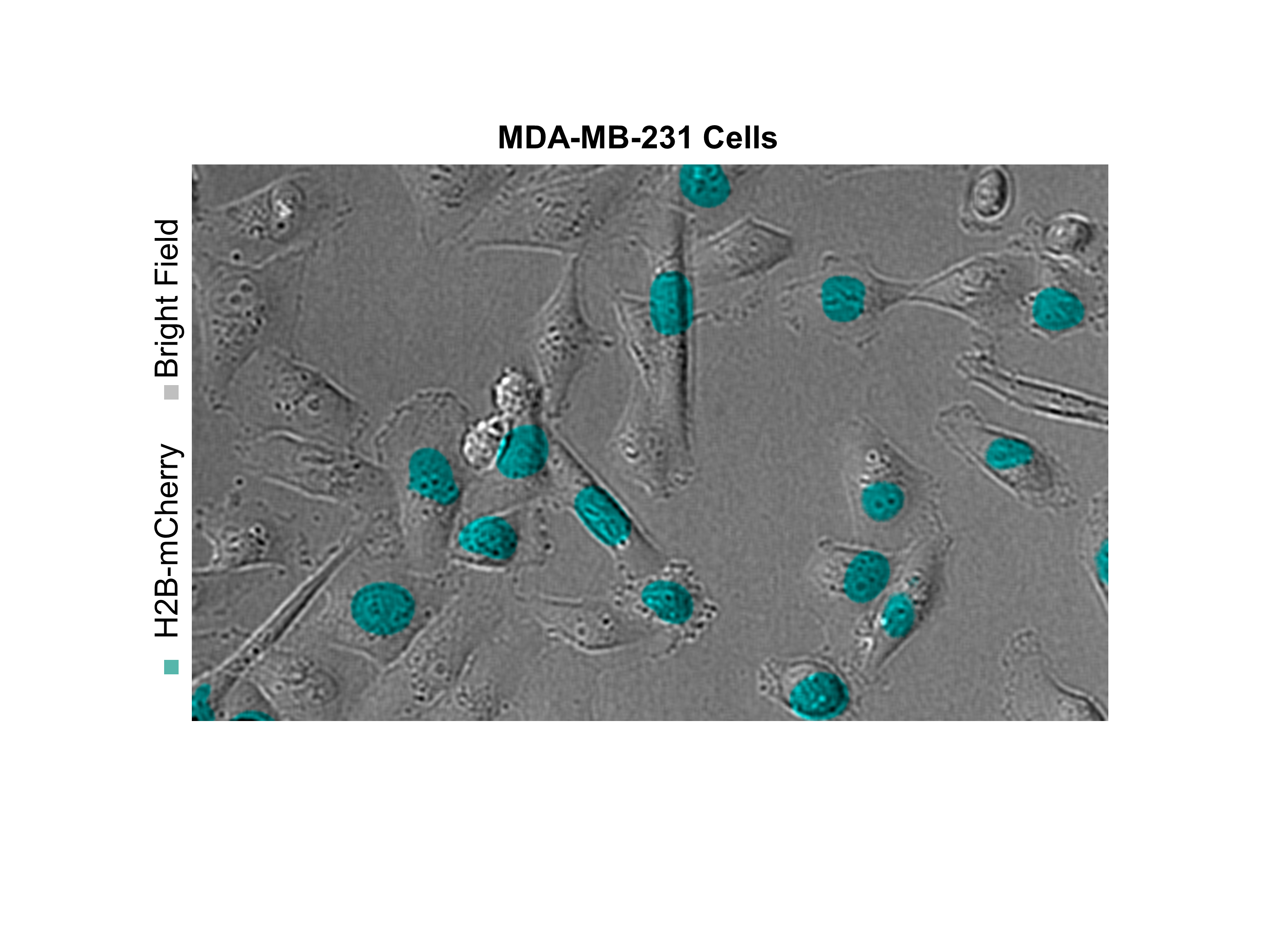

Supplement: Figure S4 — MDA-MB-231 cells maintain physical contact with their nearest neighboring cell. Brightfield microscopy of EGF treated MDA-MB-231 cells reveals that nearest neighboring cells have physical contact with each other. (TIF) [file pone.0082444.s007.tif]
